# Primary *Mycobacterium avium* Enteritis in a Patient Infected with Human Immunodeficiency Virus

**DOI:** 10.1590/0037-8682-0334-2021

**Published:** 2021-08-20

**Authors:** Chee Yik Chang

**Affiliations:** 1 Hospital Sultanah Aminah, Department of General Medicine, Johor, Malaysia.

**FIGURE 1:**
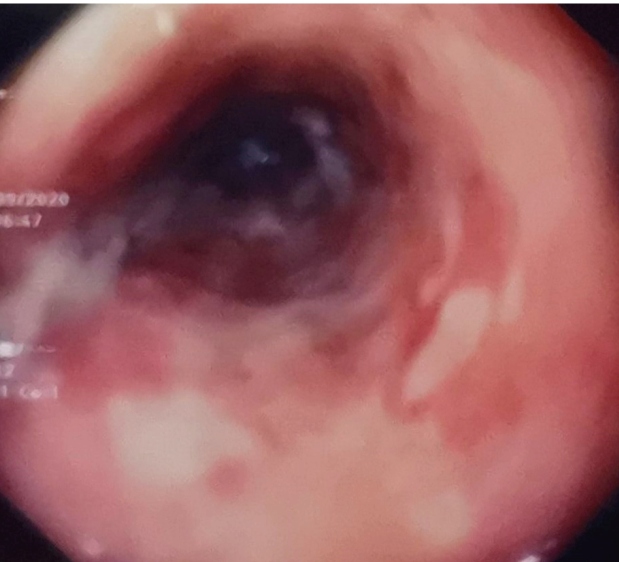
Scattered white plaques with areas of raw mucosa along the esophagus.

**FIGURE 2:**
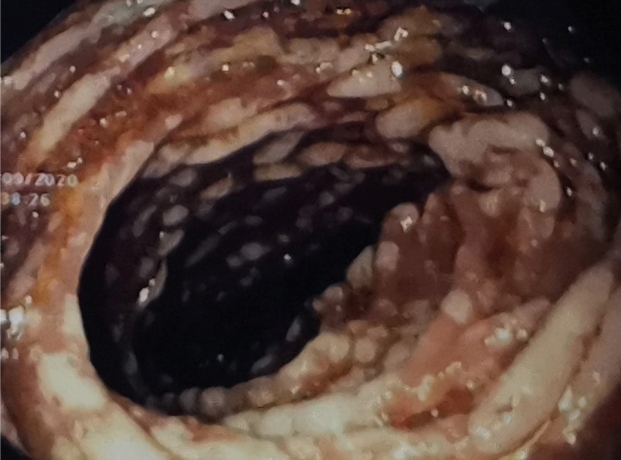
Extensive white mucosal plaques in the duodenum.

A 54-year-old man with advanced human immunodeficiency virus (HIV) disease (CD count=5 cells/mm^3^) was hospitalized for odynophagia and weight loss persisting since one month. He had cachexia and oral thrush. Upper endoscopy was performed for evaluating odynophagia, which showed scattered white plaques with areas of raw mucosa along the esophagus, suggesting esophageal candidiasis (Figure 1) and extensive white mucosal plaques in the duodenum (Figure 2). Histopathological examination of the small intestinal biopsy specimens revealed numerous acid-fast bacilli, confirming *Mycobacterium* spp. infection. Periodic acid-Schiff staining was negative for fungal elements. Blood culture was negative, whereas a culture of the small intestine biopsy revealed *Mycobacterium avium*. Hence, a diagnosis of primary *M. avium* enteritis was made. He was started on antiretroviral therapy along with combination therapy for *M. avium* complex (MAC) disease consisting of rifampicin, ethambutol, ciprofloxacin, and clarithromycin. 

MAC disease is an important acquired immune deficiency syndrome (AIDS)-defining opportunistic infection that typically occurs in HIV-infected individuals with CD4 counts of <50 cells/mm^3^. The availability of effective antiretroviral therapy and chemoprophylaxis has markedly reduced the incidence of disseminated MAC infection and improves the survival of the affected individuals^1^. MAC colonization in the gastrointestinal tract is associated with a risk of MAC bacteremia of 60% within one year compared with those without gastrointestinal involvement^2^. Gastrointestinal MAC infection may be the first manifestation of AIDS-associated opportunistic infection; hence, clinicians should be alert regarding the endoscopic findings of gastrointestinal MAC disease. Prompt initiation of anti-MAC therapy and antiretroviral therapy may reduce morbidity and mortality.
